# How well do cancer survivor self-classifications of anxiety, depression and stress agree with a standardised tool? Results of a cross-sectional study

**DOI:** 10.1371/journal.pone.0222107

**Published:** 2019-09-20

**Authors:** Mariko Carey, Jamie Bryant, Alison Zucca, Alix Hall, Alice Grady, Sophie Dilworth, Kerry Peek

**Affiliations:** 1 Health Behaviour Research Collaborative, School of Medicine and Public Health, Faculty of Health and Medicine, University of Newcastle, Callaghan, NSW, Australia; 2 Priority Research Centre for Health Behaviour, University of Newcastle, Callaghan, NSW, Australia; 3 Hunter Medical Research Institute, New Lambton Heights, NSW, Australia; 4 Hunter New England Local Health District, Population Health, Wallsend, NSW, Australia; University of Auckland, NEW ZEALAND

## Abstract

**Background:**

There is poor uptake of psychosocial interventions offered to people with cancer who record high scores on distress screening scales. Intervention uptake may be influenced by a mismatch between consumer (bottom-up) and professional (top-down) paradigms of wellbeing. The current research aims to compare cancer survivors’ ‘self-judgements’ about their levels of anxiety, depression and stress, to classifications derived via a professional-driven measure, the Depression, Anxiety and Stress Scale (DASS-21).

**Methods:**

A cross-sectional study was undertaken with haematological cancer survivors recruited from three population-based cancer registries in Australia. Consenting participants were mailed a questionnaire package; and non-responders received a second questionnaire package after 3-weeks and a reminder call after 6-weeks. The consumer-driven perspective was assessed via three separate single items asking survivors to self-classify their levels of anxiety, depression and stress over the past week on a scale from ‘normal’ to ‘extremely severe’. The professional-driven classification was assessed via the DASS-21. Kappa statistics were used to assess agreement between consumer- and professional-driven measures.

**Results:**

Of 2,971 eligible haematological cancer survivors, 1,239 (42%) provided written consent and were mailed a questionnaire package. Of these, 984 (79%) returned a completed questionnaire. The simple kappa for agreement between the DASS-21 and self-classified measures for anxiety was 0.47 (95% CI: 0.39 to 0.54, p<0.0001). The weighted kappa for agreement between the DASS-21 and self-classified measures of depression was 0.60 (95% CI: 0.53 to 0.67, p<0.0001) and for measures of stress was 0.51 (95% CI: 0.44 to 0.59, p<0.0001).

**Conclusions:**

Moderate agreement between self-classification and professional-driven assessments was found. The value of screening is predicated on the assumption that those with identified needs will be offered and take up services that will benefit them. Our results suggest that to improve the utility of distress screening it may be important to include assessment of survivor views about their symptoms.

## Introduction

The effectiveness of psychological distress screening interventions in cancer settings remains unclear [1–3]. In a systematic review of 24 intervention studies, evaluating the effectiveness of screening for distress and unmet needs in cancer settings, most studies (71%) reported benefits on quality of care delivery including clinician behaviour (e.g. referrals for psychological care; communication). However, only one-third (n = 8) of screening interventions resulted in any direct benefits on patient well-being (such as distress, anxiety, depression, quality of life). In contrast, another systematic review [2], which applied a stringent inclusion criteria, found only one study that could evaluate the impact of distress screening alone on well-being. This study did not show any impact of distress screening on patient psychological morbidity [4].

Poor uptake of psychosocial interventions by people with cancer who record high scores on distress scales may be a contributor to the modest effectiveness of screening interventions. For example, in a sample of 331 people attending oncology outpatients with significant distress, 71% declined help for any psychological services offered [5]. Similarly, in samples of 4000 [6] and 227 [7] people with cancer who had elevated emotional distress (as determined by the distress thermometer cut-off ≥ 5), 57% did not report a need for psychological support [6]; and 57% did not want a referral for psychological support [7]. Common reasons for patients declining help include: a preference for self-management; reported to be already receiving help; a belief that the distress is not severe enough to warrant assistance; inconvenient timing of the service offered; and a belief that the service was unnecessary or would not be helpful [5, 8]. These barriers to distress screening also reflect challenges that prevent high quality of psychosocial care in general [3, 9].

Uptake of psychosocial interventions may be influenced by a mismatch between consumer (bottom-up) and professional (top-down) paradigms of wellbeing. The fundamental difference between these approaches is determining who is the expert in judging patient well-being [10]. In the traditional professional-driven approach, clinically defined criteria or thresholds are used to determine the need for help (i.e. caseness). The health professional is the expert and their judgement is deemed the gold standard. Measures of anxiety and depression, such as the Hospital Anxiety and Depressions Scale (HADS) and the Depression, Anxiety and Stress Scale (DASS-21) are examples of tools used in this approach. Alternatively, in a consumer–driven approach, patients play the expert role in defining and classifying well-being with reference to their own experiences, preferences, values and needs [10]. Consumer-driven approaches may be particularly relevant to screening for distress. Salmon [11] has noted that unlike biomedical screening which identifies a biomarker of disease which would remain unknown to the patient, distress screening identifies only those concerns that the patient has identified and is willing to disclose to the health care professional. Therefore, consumer perceptions of their level of distress and need for help are crucial to enable the provision of high quality psychosocial care.

This has led to a call for a refocussing the distress screening agenda to explore what contextual information, including information on patients’ own views about their level of wellbeing and need for help, may improve the utility of screening [11]. Mackenzie and colleagues’ study [12] of 304 people undergoing radiotherapy demonstrated that consumer self-assessed levels of anxiety and depression have only moderate agreement with classified levels of anxiety and depression using the HADS. Similarly, there was some evidence of discordance between self-assessments and levels of anxiety and depression classified using the HADS among people with advanced cancer [13] and medical oncology patients [14]. There is a need to examine whether these findings are generalizable when using other types of distress tools, including those which measure constructs other than anxiety and depression. The current research aims to compare cancer survivors’ self-judgements about their levels of anxiety, depression and stress to their classification as anxious, depressed or stressed using a standardised instrument, the DASS-21 [15].

## Methods

### Design

This was a sub-study of a larger, cross-sectional survey study assessing the psychosocial wellbeing and unmet needs of Australian haematological cancer survivors recruited from five state, population-based cancer registries [16]. Human research ethics approval was obtained from the University of Newcastle Human Research Ethics Committee (2009–032); Cancer Institute NSW Population and Health Service Research Ethics Committee (2009/05/157); Tasmanian Health and Medical Research Human Research Ethics Committee (H0010603); Cancer Council Victoria’s Human Research Ethics Committee (HREC 0913); Department of Health Western Australia Human Research Ethics Committee (2009/11); and Queensland Health Research Ethics and Governance Unit (RD002733). This sub-study consisted of a survey module completed by haematological cancer survivors recruited from three of the registries.

### Survivor sampling and recruitment

Adult survivors, aged 18 years and over at the time of the study, and diagnosed with a haematological cancer including, non-Hodgkin lymphoma, leukaemia, myeloma, and other lymphomas were recruited from three state cancer registries. In line with the National Cancer Institute definition, people were considered to be survivors from the time of cancer diagnosis until death [17]. Survivors were classified as residing in urban or rural locations based on Accessibility and Remoteness Index of Australia (ARIA +) [18]. Postcodes classified by ARIA+ as major cities and inner regional were classified as “urban”; while with an ARIA+ classification of outer regional, remote and very remote locations were classed as “rural” [18]. Different sampling approaches for rural versus urban survivors were employed because the larger study [16] from which this sub-study was derived aimed to compare specific outcomes of rural and urban survivors. Therefore, to ensure adequate numbers of rural participants, all eligible rural survivors were invited to participate. Given the larger number of urban survivors, a random sample of eligible urban survivors were invited to participate. It is a requirement of some registries that cancer survivors are only contacted once about research, and as a result survivors who had been previously contacted were excluded prior to sampling at registries where this policy was implemented.

Registries wrote to haematological cancer survivors’ notifying clinicians and asked them to contact the registry within one month if the survivor should not be approached. Eligible survivors were then contacted by the registries and sought written consent to pass on their contact details to the research team. Consenting survivors were mailed a questionnaire, information statement and reply-paid envelope. A second questionnaire package was sent to consenting non-responders after 3 weeks, and a reminder telephone call was made 6 weeks following the mailing of the study survey.

### Measures

#### Depression, Anxiety and Stress Scale—21 (DASS-21)

[15]. This is a self-report ‘top-down’ or ‘professional driven’ measure of depression (7 items), anxiety (7 items) and stress (7 items). Respondents are asked to indicate the degree to which they have experienced each item over the past week using a 4 point likert scale (0 = not at all; 3 = very much so) [15]. A total score is calculated for each of the three subscales by summing all items in a subscale and multiplying by two, with higher scores representing higher levels of the construct [15]. The DASS-21 has evidence of construct and concurrent validity and internal consistency in clinical and non-clinical samples [19–21].

#### Single-item self-classification of anxiety, depression and stress

Three separate single items to assess survivor’s self-classified levels of anxiety, depression and stress respectively were employed for a consumer-driven perspective. For example, participants were asked to indicate their level of depression over the past week using the following response options: “normal,” “mild,” “moderate,” “severe” and “extremely severe.” The response scale used for this question was designed to reflect the recommended cut-points used to score the DASS-21. Single-items are available at S1 File.

#### Explanatory variables

For survivors who provided consent, the registry provided the researchers with details regarding their: age at diagnosis, sex, date of diagnosis, haematological cancer type and postcode of residence. Other demographic and disease characteristics were collected via survivor self-report, including: marital status, employment, education level, cancer treatments received, and current treatment for anxiety and depression.

### Statistical analysis

Frequencies and percentages were calculated for all explanatory variables. DASS-21 subscales were scored according to the developers’ recommendations [15]. For the depression subscale, normal symptoms are indicated by scores 0–4; mild by scores 5–6; moderate by scores 7–10; severe by scores 11–13 and extremely severe by scores of 14 or more. For the anxiety subscale, normal symptoms are indicated by scores of 0–3; mild by scores of 4–5; moderate by scores of 6–7; severe by scores of 8–9; and extremely severe by scores of 10 or more. For the stress scale, normal symptoms are indicated by scores 0–7; mild by scores 8–9; moderate by scores 10–12; severe by scores 13–16; and extremely severe by scores of 17 or more [15].

Kappa statistics were used to assess the agreement between patients’ DASS-21 classifications and a single-item for self-classified anxiety/depression/stress. DASS-21 and single-item measurements were categorised as normal/mild or moderate/severe/extremely severe. Participants with missing data for either the single item self-classification or the relevant DASS-21 subscale were excluded. 95% Confidence intervals and p-values from the Z-test (with the null hypothesis of kappa = 0) were calculated. Statistical analyses were programmed using SAS v9.4 (SAS Institute, Cary, North Carolina, USA).

## Results

A total of 2,951 eligible haematological cancer survivors were contacted by the three registries, of which 1,239 (42%) provided written consent to have their contact details released to the researchers and were thus mailed a questionnaire package. Of these, 984 (79%) returned a completed survey. The sociodemographic, disease and treatment characteristics of study participants are presented in Table 1. For the larger main study, there were statistically significant differences between participants and non-participants in terms of age at diagnosis, location of residency, cancer type and registry from where survivors were recruited from [16].

**Table 1:** Participant sociodemographic, disease and treatment characteristics.

Characteristic	Level descriptor	Total (N = 984)^a^	
		n	%
Age	18 to 39	92	(11)
	40 to 49	90	(10)
	50 to 59	255	(29)
	60+	435	(50)
Gender	Female	368	(42)
	Male	504	(58)
Location of residence	Rural	137	(15)
	Urban	784	(85)
Marital status	Single	76	(7.8)
	Married or defacto	752	(77)
	Separated or divorced	84	(8.6)
	Widowed	63	(6.5)
Highest level of education	High school or below	369	(38)
	Vocational or other	305	(32)
	University	290	(30)
Employment	Full, part time, or paid leave	380	(39)
	Unpaid leave, unemployed or other	592	(61)
Current Anxiety or Depression Treatment	Yes	163	(17)
	No	777	(83)
Time since diagnosis	≤12 months	6	(0.7)
	13 to 24 months	68	(7.8)
	25 to 36 months	116	(13)
	37 to 48 months	186	(21)
	49 to 60 months	304	(35)
	61+	192	(22)
Cancer type	Leukaemia	126	(14)
	Myeloma	129	(15)
	Non Hogkins Lymphoma	514	(59)
	Other	103	(12)
Currently receiving treatment for cancer	No	23	(2.3)
	Yes	961	(98)

^a^Numbers may not sum to 984 due to missing data.

Cross tabulation of level of anxiety as measured by the DASS-21 anxiety subscale and the single-item self-classification of anxiety is presented in Table 2 (n = 943, 41 missing). Of those classified as having normal/mild anxiety using the DASS-21, 93% (n = 723) were similarly classified using the single-item self-classification. Of those who were classified as having moderate/severe/extremely severe symptoms of anxiety using the DASS, only 50.6% (n = 84) were similarly self-classified as having moderate/severe/extremely severe anxiety using the single-item self-classification. The simple kappa for the agreement between the DASS subscale and single-item self-classification of anxiety was 0.47 (95% CI: 0.39 to 0.54, p<0.0001), and raw agreement was 85.6%.

**Table 2:** Agreement between levels of anxiety as self-classified by a single-item and the DASS-21 anxiety subscale (n = 943).

	DASS-21 Anxiety	
**Single-item**	**Normal/Mild (%)**	**Moderate/Severe/ Extremely severe (%)**
**Normal/Mild**	723 (93.0)	82 (49.4)
**Moderate/Severe/ Extremely severe**	54 (7.0)	84 (50.6)

Cross tabulation of level of depression as measured by the DASS-21 depression subscale and the single-item self-classification of depression measure is presented in Table 3 (n = 946, 38 missing). Of those classified as having normal/mild depression using the DASS-21, 96.0% (n = 764) were similarly classified using the single-item self-classification. Of those who were classified as having moderate/severe/extremely severe depression using the DASS-21, 59.3% (n = 89) were similarly self-classified. The weighted kappa for the agreement between the DASS subscale and single-item self-classification of depression was 0.60 (95% CI: 0.53 to 0.67, p<0.0001), and raw agreement was 90.2%.

**Table 3:** Agreement between levels of depression as self-classified by a single-item and the DASS-21 depression subscale (n = 946).

	DASS-21 Depression	
**Single-item**	**Normal/Mild (%)**	**Moderate/Severe/ Extremely severe (%)**
**Normal/Mild**	764 (96.0)	61 (40.7)
**Moderate/Severe/ Extremely severe**	32 (4.0)	89 (59.3)

Cross tabulation of level of stress as measured by the DASS-21 stress subscale and the single-item self-classification of stress is presented in Table 4 (n = 943, 41 missing). Of those classified as having normal/mild stress using the DASS-21, 89.3% (n = 746) were similarly self-classified. Of those classified as having moderate/severe/extremely severe stress using the DASS-21, 75% (n = 81) were similarly self-classified using the single-item measure. The weighted kappa for the agreement between the DASS subscale and self-classification of stress was 0.51 (95% CI: 0.44 to 0.59, p<0.0001), and raw agreement was 87.7%.

**Table 4:** Agreement between levels of stress as self- classified by a single-item and the DASS-21 stress subscale (n = 943).

	DASS-21 Stress	
**Single-item**	**Normal/Mild (%)**	**Moderate/Severe/ Extremely severe (%)**
**Normal/Mild**	746 (89.3)	27 (25.0)
**Moderate/Severe/ Extremely severe**	89 (10.7)	81 (75.0)

## Discussion

To our knowledge this is the largest study to explore agreement between self-classified levels of psychological morbidity and the DASS-21 for assessing level of psychological morbidity amongst haematological cancer survivors. Our findings confirm those of a prior study [12] which demonstrated moderate agreement between self-classified anxiety and depression and classification according to a standardised scale. The present study, however, offers new information by demonstrating moderate agreement for self-classified stress and a standardised scale. The Kappa coefficient for the anxiety self-classification and the subscale was lowest at 0.4, while the Kappa for the depression self-classification and subscale was the highest at 0.6. The higher Kappa for self-classification of depression, however, appeared to be mainly due to a high raw agreement between the self-classification and subscale score for those reporting normal and mild symptoms.

Raw agreement between each subscale score and the single-item self-classification for normal and mild levels of symptoms was high, ranging from 89.3% for stress to 95.9% for depression. Raw agreement for moderate to extremely severe levels of symptoms was high for the stress subscale and its self-classification (75%); however, only moderate agreement for the anxiety (51%) and depression (59%) subscales and self-classification. These results suggest that only self-classifications of stress were likely to provide similar information to that obtained from a standardised tool in terms of identifying people with elevated symptoms. This may reflect that anxiety and depression have a greater associated stigma than stress, and therefore, participants experiencing symptoms of depression or anxiety may be reluctant to label these as severe. Alternatively, it may reflect that the depression and anxiety subscales do not identify contextual factors that inform survivors’ self-assessments about what high levels of symptoms of depression or anxiety mean. For example, survivors may normalise symptoms of worry, sadness as being normal reactions to a cancer diagnosis, or physical discomfort associated with the disease or its treatment [22], and may expect their psychological symptoms to be transient and self-resolving. These factors may influence the way in which elevated symptoms of anxiety and depression are viewed by and labelled by survivors.

### Clinical implications for distress screening

The value of screening is predicated on the assumption that those with identified needs will be offered and take up services that will benefit them. Our results suggest that to improve the utility of distress screening, it may be important to include assessment of survivor views about their symptoms. Moderate agreement between self-classification and standardised assessments as demonstrated in our study, suggests that referrals based solely on screening results are unlikely to result in high levels of uptake of services. Mackenzie et al.’s work [12], for example, has shown that self-assessed needs are more strongly associated with preference for professional help with anxiety or depression than scores on a standardised measure such as the HADS. Where discordance between standardised tools and survivor self-classifications exist, an opportunity is presented for clinicians to explore reasons for discordance, and provide care that is more responsive to the survivor’s needs.

Reliance solely on results of standardised tools also means that some people who self-classify as highly distressed, and are not classified as distressed on a standardised measure, could miss out on being offered services that may benefit them. This may reflect the inability of tools focussing on issues like anxiety and depression to capture other concerns of individuals, who may benefit from intervention.

To improve care delivery, it may be necessary for researchers and clinicians to broaden their focus beyond top-down diagnostic distress screening [11]. When top-down and bottom-up perspectives align, care delivery may continue as usual. However, when they do not align, there may be a need for health professionals to explore, with patients, the possible reasons for this. Explicit discussion of the mismatch between these perspectives may allow for issues such as lack of understanding of symptoms which comprise depression or anxiety or concerns about stigma associated with mental illness to be addressed. Conversely, discussion may highlight contextual factors which suggest that symptoms are temporary or resolving, or which aid in understanding the triggers for the symptoms experienced. These factors, as well as the severity of symptoms reported, may provide guidance when making decisions about whether treatments or services should be recommended and if so, which ones.

### Limitations

The population-based sampling used in this study was a strength as it allowed inclusion of a broad range of haematological cancer survivors. The low response rate, while similar to that achieved by other studies which have recruited via cancer registries [23, 24], means that consent bias cannot be ruled out. Therefore, it is unlikely that results are generalizable to all haematological cancer survivors. Finally, no definitions of depression, anxiety or stress were provided to participants. As such, interpretation of these concepts are likely to vary among participants.

## Conclusions

This paper details the level of agreement between single-item self-classifications and a standardised self-report tool for assessing psychological morbidity amongst haematological cancer survivors in Australia. The tested single-item self-classifications had moderate agreement with DASS-21 subscales for anxiety, depression and stress. Survivor self-classification of levels of psychological morbidity may be a useful adjunct to standardised assessments, and may assist in understanding which survivors are likely to accept referrals to support services.

## Supporting information

S1 File Instructions and single-items used in this study. (DOCX)
