# Supplementary material for: How well do cancer survivor self-classifications of anxiety, depression and stress agree with a standardised tool? Results of a cross-sectional study
Source: PLoS One. 2019 Sep 20;14(9):e0222107. doi: 10.1371/journal.pone.0222107 (PMC6754128; doi:10.1371/journal.pone.0222107)
Supplement: S1 File — (DOCX) [file pone.0222107.s001.docx]

**Supporting Information**

**Instructions and single-items used in this study**

Sometimes people who have been touched by cancer **feel distressed and upset.** We want to know about your levels of distress **in the last week**. Even if you have never experienced any depression, anxiety or stress, we are still very interested in hearing about your experiences.

**Please circle the response which most closely describes your feelings over the past week.**

|  | | **Normal** | **Mild** | **Moderate** | **Severe** | **Extremely severe** |
| --- | --- | --- | --- | --- | --- | --- |
| **Q1** | **Over the past week my level of depression has been:** | 1 | 2 | 3 | 4 | 5 |
| **Q2** | **Over the past week my level of anxiety has been:** | 1 | 2 | 3 | 4 | 5 |
| **Q3** | **Over the past week my level of stress has been:** | 1 | 2 | 3 | 4 | 5 |
|  | | | | | | |
